# Estimation of brain amyloid accumulation using deep learning in clinical [^11^C]PiB PET imaging

**DOI:** 10.1186/s40658-023-00562-7

**Published:** 2023-07-14

**Authors:** Claes Nøhr Ladefoged, Lasse Anderberg, Karine Madsen, Otto Mølby Henriksen, Steen Gregers Hasselbalch, Flemming Littrup Andersen, Liselotte Højgaard, Ian Law, for the Alzheimer’s Disease Neuroimaging Initiative

**Affiliations:** 1 Department of Clinical Physiology and Nuclear Medicine, Rigshospitalet, University of Copenhagen, Blegdamsvej 9, 2100 Copenhagen, Denmark; 2 Danish Dementia Research Centre, Rigshospitalet, University of Copenhagen, Blegdamsvej 9, 2100 Copenhagen, Denmark

**Keywords:** AI, Alzheimer’s disease, Amyloid, Automatic diagnosis, Convolutional neural network, Decision support, Deep learning, Dementia, PET, Stratification

## Abstract

**Introduction:**

Estimation of brain amyloid accumulation is valuable for evaluation of patients with cognitive impairment in both research and clinical routine. The development of high throughput and accurate strategies for the determination of amyloid status could be an important tool in patient selection for clinical trials and amyloid directed treatment. Here, we propose the use of deep learning to quantify amyloid accumulation using standardized uptake value ratio (SUVR) and classify amyloid status based on their PET images.

**Methods:**

A total of 1309 patients with cognitive impairment scanned with [^11^C]PIB PET/CT or PET/MRI were included. Two convolutional neural networks (CNNs) for reading-based amyloid status and SUVR prediction were trained using 75% of the PET/CT data. The remaining PET/CT (*n* = 300) and all PET/MRI (*n* = 100) data was used for evaluation.

**Results:**

The prevalence of amyloid positive patients was 61%. The amyloid status classification model reproduced the expert reader’s classification with 99% accuracy. There was a high correlation between reference and predicted SUVR (*R*^2^ = 0.96). Both reference and predicted SUVR had an accuracy of 97% compared to expert classification when applying a predetermined SUVR threshold of 1.35 for binary classification of amyloid status.

**Conclusion:**

The proposed CNN models reproduced both the expert classification and quantitative measure of amyloid accumulation in a large local dataset. This method has the potential to replace or simplify existing clinical routines and can facilitate fast and accurate classification well-suited for a high throughput pipeline.

**Supplementary Information:**

The online version contains supplementary material available at 10.1186/s40658-023-00562-7.

## Background

Alzheimer’s disease (AD) is a neurodegenerative disease characterized by parenchymal amyloid-β (Aβ) deposits [1], which can be investigated noninvasively by amyloid positron emission tomography (PET). Several clinically implemented PET tracers (e.g., [^18^F]AV-45 ([^18^F]Florbetapir) and [^11^C]Pittsburg-compound-B ([^11^C]PiB)), that bind with high affinity to amyloid plaques, allow for the inference of amyloid status in dementia and the early diagnosis of probable AD [2, 3]. The development of high throughput fast and accurate data analysis strategies to detect or exclude the presence of Aβ plaques in PET scans could be an important tool in patient selection and monitoring for amyloid directed treatment presently FDA approved or in the pipeline [4, 5].

Amyloid PET imaging is performed as a clinical qualitative reading with specific criteria for amyloid PET image interpretation, that may differ among available radiotracer as defined in recent imaging guidelines [6]. The inter-rater variability is generally low, as demonstrated by Yamane et al. [7] who found near perfect agreement between three raters (93.2% for binary criteria). The clinical interpretation is often supported by semi-quantitative metrics for global cortical uptake, where tracer binding is assessed in a standard set of cortical regions normalized by a reference region, also known as the standardized uptake value ratio (SUVR) [8, 9]. Ideally, brain parcellation using a structural image (e.g., MRI) is performed. However, this may be impractical in routine clinical use, as MRI might not be available or was performed in unstandardized protocols, which can require laborious data management and quality assurance to secure adequate brain coverage, and detect inaccurate co-registration or segmentation [10, 11].

Deep learning, a subset of machine learning, is increasingly used in medicine and for assessment of brain disorders [12]. Most studies on disease detection focus on AD, partly due to the availability of the large public dataset Alzheimer’s Disease Neuroimaging Initiative (ADNI). Recently, end-to-end solutions based only on PET images as input have demonstrated high accuracy for classifying amyloid burden through training of a convolutional neural network (CNN) [10, 11, 13, 14]. Kim et al. trained a CNN to predict the SUVR value from 850 [^18^F]Florbetapir scans extracted from ADNI that showed 94% accuracy when using a predefined SUVR threshold for amyloid test positive/negative [14], and Reith et al. [10] obtained a 95% accuracy when using 2066 [^18^F]Florbetapir ADNI scans as input to a CNN pre-trained on ImageNet (a dataset of natural images) [10].

However, the reported accuracies might not be representative of local performance, since PET scans in ADNI do not represent the variation found in a clinical cohort caused by deviations from scan protocols, incl dose, patient motion, and acquisition delay or period. Furthermore, ADNI consists of a highly selected cohort consisting of healthy controls, mild cognitive impairment (MCI) and AD patients, and recently also subjective cognitive decline (SCD), and excludes other patients that are referred to amyloid PET in a clinical setting. This could be patients where MRI cannot be performed, because of metal implants or patients with suspected competing pathology that may manifest as cognitive deficits, e.g., structural brain lesions or deformations caused by as stroke, trauma, or normal pressure hydrocephalus (NPH), and other neurological or psychiatric conditions.^1^ Furthermore, there are a significant proportion of other non-AD neurodegenerative conditions with cognitive deficits, such as frontal temporal lobar dementia (FTLD), and atypical parkinsonism, both with subgroups of specific clinical and imaging manifestations, that are not represented in ADNI.

In this study, we aimed to develop an automated classification model for assessing amyloid status as well as a regression model for predicting the amyloid SUVR from a [^11^C]PiB PET image in a mixed memory clinic cohort. The amyloid status is compared against a visual clinical interpretation of the images by an expert reader, and the predicted SUVR is compared against the semi-quantitative reference. Furthermore, we wished to assess the added value of an automated classification model vs. other simplified methods such as the SUVR threshold. Our study took inspiration from several novel studies in the field [10, 13, 14], but deviates from these by utilizing a large local dataset for training and evaluation.

## Materials and methods

### Patients

We identified all patients undergoing [^11^C]PiB PET using PET/CT or PET/MRI between March 2010 and October 2020 at Rigshospitalet, Copenhagen, Denmark. All patients were referred from a memory clinic specialist after standard clinical evaluation for dementia with the suspicion of an underlying amyloid pathology according to appropriate use criteria [15]. Patients with a non-interpretable scan and patients referred for suspected cerebral amyloid angiopathy were excluded. Thirteen hundred and nine patient examinations were included in this study. The patients had an average age at the examination time of 69 ± 9 years (range: 39–89 years). Test set data were independent from the training data; we divided the subsets according to scanner type and competing structural pathology on current or previous CT or MRI, see Table 1. The training set (*n* = 872) consisted of 75% of the PET/CT examinations randomly sampled from the subjects that did not have any suspected competing pathology. The training set was used to develop both our automatic classification models and determine the SUVR threshold. The methods were mainly evaluated using two test sets consisting of the hold-out PET/CT examinations (*n* = 300) and the hold-out PET/MRI examinations (*n* = 100). In a separate evaluation, we evaluated the methods’ ability to overcome challenging cases with significant co-morbidity including patients with previous brain trauma or surgery, brain infarcts, hemorrhages, meningiomas, cysts, or multiple sclerosis (*n* = 37).

**Table 1:** Patient demographics for each dataset

Cohort	Age [yrs]	Dose [MBq]	Scan start p.i. [min]	*N*
Training set	69 ± 8 (40–89)	367 ± 111 (27–754)	42 ± 4 (32–63)	872
*Hold-out tests*				
PET/CT	69 ± 9 (40–87)	347 ± 112 (51–716)	42 ± 4 (32–58)	300
PET/MRI	68 ± 9 (39–86)	422 ± 109 (170–709)	43 ± 5 (35–62)	100
Competing pathology	68 ± 11 (35–81)	435 ± 104 (227–702)	42 ± 3 (38–52)	37*

*p.i.*, post-injection

*n = 30 examined on PET/CT and *n* = 7 on PET/MRI

### Image acquisition

The patients were injected with 368 ± 113 MBq (range: 27–754 MBq) [^11^C]PiB and was examined on either a PET/CT or a PET/MRI scanner 42 ± 4 min (range: 32–63 min) post-injection (p.i.) of the radiotracer. PET data were acquired for 20 min (*n* = 1077) or 30 min (*n* = 232) over a single bed-position. Examinations were originally reconstructed following clinical protocol used at our institution at the time, which is individually tailored for each scanner. Variations include reconstructed scan period, usually 40–60 and 40–70 min p.i., but could be delayed because of scan failure or patient compliance, matrix size, voxel spacing, and post-filtering (3 mm, 4 mm, and 5 mm Gaussian). Details are given for each of the four different scanners used in Additional file 1: Table S1. We used the original reconstructed PET images extracted from our imaging archive system.

The scan protocol included a low-dose CT image (120 kVp, 30 or 40 mAs, 512 × 512 × 74 matrices, 0.59 × 0.59 × 3 mm^3^ voxels) for PET/CT and T1-weighted (T1w) MPRAGE (512 × 512 × 192 matrices, 0.49 × 0.49 × 1 mm^3^ voxels) for PET/MRI. Same-day low-dose CT was acquired for all PET/MRI examinations and used for attenuation correction of the PET signal following a co-registration to the T1w MPRAGE [16].

### Image pre-processing

The PET images were first transferred into common MNI space using the associated anatomical image (CT or MRI). For PET/CT examinations, we first smoothed the low-dose CT with a 1 mm kernel and clamped the values to lie between 0 and 100 HU using fslmaths [17], followed by skull stripping using FSL-BET [18]. For the PET/MRI examinations skull stripping was performed on the T1w MPRAGE using HD-BET [19]. Skull stripped anatomical images were registered to MNI space using affine registration only (RegAladin, niftyreg [20]). The PET images were subsequently warped to MNI space using the same affine transformation matrix, followed by a brain extraction using the brain mask obtained from skull stripped CT or MRI. The resulting resampled PET volumes had a matrix size of 256 × 256 × 256 with 1 mm^3^ isotropic voxels. PET intensities were normalized to 0–1 by dividing by the 95% quantile value and clipping the maximum value at 1 for each brain extracted PET image in MNI space.

### Visual interpretation of amyloid PET and SUVR calculation

The visual reading was performed by a board-certified nuclear medicine physician (IL), who had all images available during reading, including any previous examinations (e.g., MRIs performed prior to the PET/CT). PET images were interpreted after registration to available MRI or CT and reviewed in coronal, sagittal and transaxial planes. PET scanning was classified on a two-point scale as amyloid negative or positive based on the criteria developed for [^18^F]Florbetapir: A positive scan had two or more brain areas each larger than a single cortical gyrus in which there were reduced or absent gray-white matter contrast or one or more areas in which gray matter uptake is intense and clearly exceeds that in adjacent white matter.

Interpretation was supported by the calculation of SUVR normalized to cerebellar gray matter. A set of cortical standard regions was developed based on FreeSurfer (6.0, http://surfer.nmr.mgh.harvard.edu) segmented MRI from 40 healthy elderly subjects of the prefrontal, orbitofrontal, parietal, temporal, anterior cingulate, posterior cingulate, precuneus cortex, and cerebellar gray matter. The regions were transferred to MNI standard space and averaged within region between subjects, and a threshold selected so that the size of each averaged region was equal to the average between subject’s volume. For analysis of patient scans, we reverse transformed the projections of standard cortical regions to native space using the individual affine MNI registration described above and obtained the median volume weighted activity values for the calculation of reference SUVR (rSUVR). Visual quality assurance of correct placement of the regions was performed for each subject based on standard printouts of 9 axial slices on PET and CT/MRI.

### End-to-end training

**Fig. 1:**
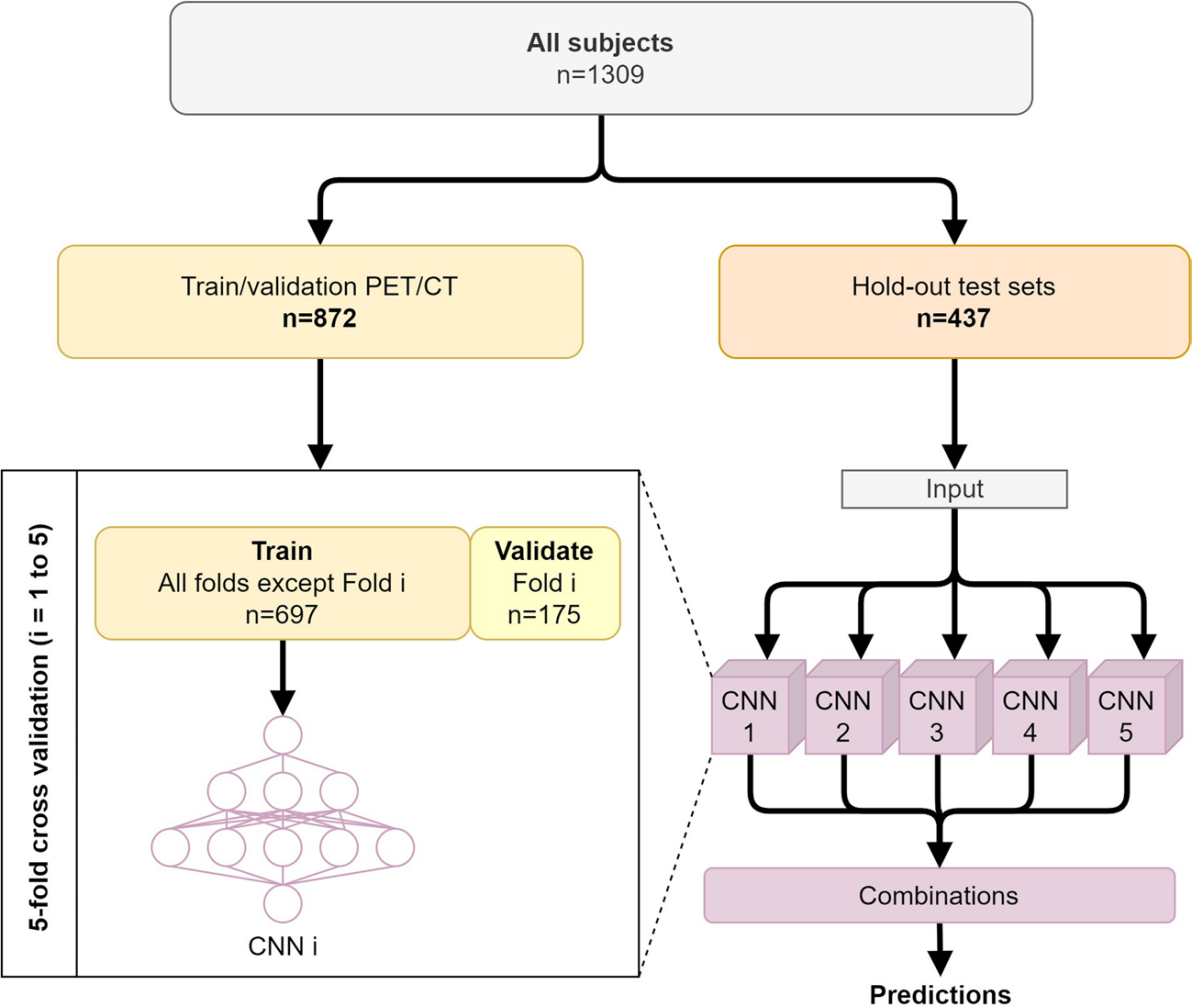
Overview of *k*-fold cross-validation training and ensemble predictions. The training/validation set consisted of 75% of the PET/CT scans. Hold-out test set was made up by the remaining 25% PET/CT scans (*n* = 300), all the PET/MRI scans (*n* = 100), and a set of challenging patients with significant co-morbidity scanned on a PET/CT (*n* = 30) or a PET/MRI (*n* = 7)

The networks were trained by fivefold cross validation using the training PET/CT subjects (*n* = 872). The hold-out test sets served as input to each of the five CNNs, and a final prediction was achieved by averaging the outputs. An overview of the data split is provided in Fig. 1. The scripts for inference and the trained model weights used in this manuscript are available at https://github.com/CAAI/amyloidAI.

**Fig. 2:**
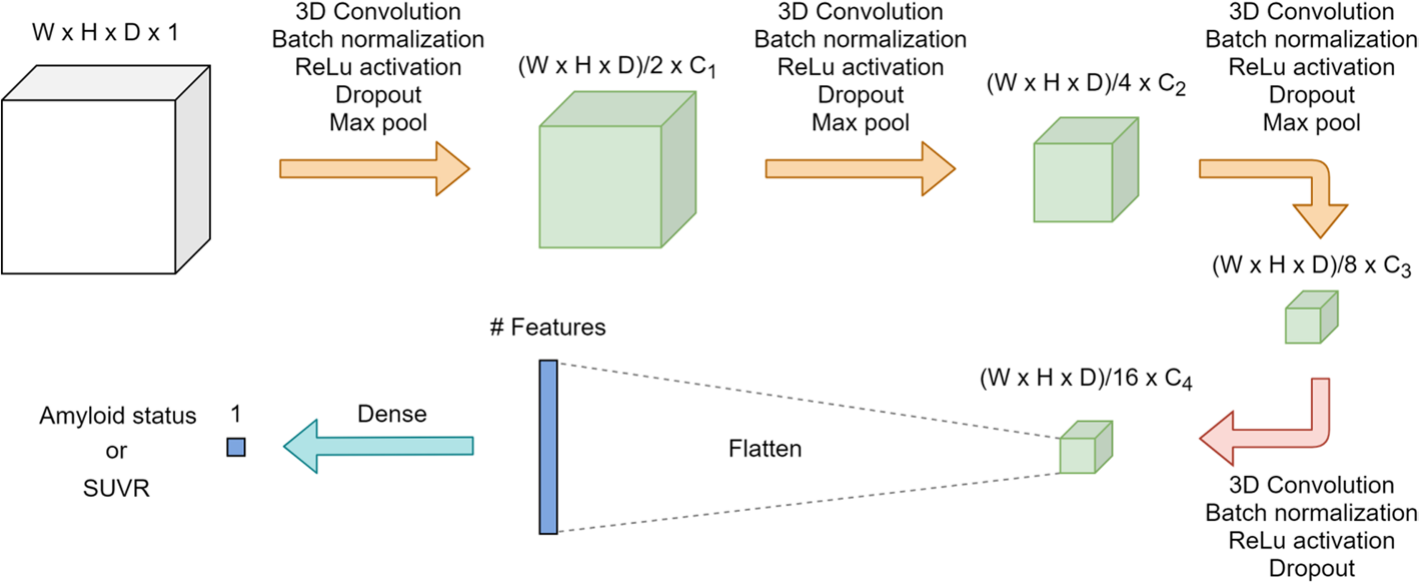
Overview of network architecture consisting of four convolutional blocks and a final dense layer. Layers in each block are indicated above each arrow. The final convolutional block (red arrow) deviates from the first three blocks (yellow arrows) by not containing a max pooling layer. The information above each box represents the size of the resulting activation maps, which are difference between the classification and regression networks. Values used are given in Additional file 1: Table S2 and S3

We trained two separate networks: the first predicting the amyloid status and the second predicting the continuous SUVR (pSUVR). Both networks were 3D CNNs with four convolutional blocks followed by a dense layer for the two networks, respectively (Fig. 2). The first three convolutional blocks consisted of a 3D convolution layer, batch normalization, rectified linear unit (ReLU) activation, dropout layer, and a max pool layer for down-sampling with pool-size and stride of 2. The last block did not contain a max pool layer. For each network individually, we performed a hyper parameter search for the optimal kernel size, number of filters, dropout fraction, and learning rate. The search space and chosen parameters are given in Additional file 1: Table S2 and S3. We used He kernel initialization and L2 penalties (l2 = 1 × 10^–4^) for kernel regularization on the convolutional layers. Both networks accepted full 3D PET volumes as input; the amyloid status network in 1 mm^3^ resolution (256 × 256 × 256), whereas the images were first downsampled to 2 mm^3^ resolution (128 × 128 × 128) for the quantitative SUVR network. The proposed networks were implemented in PyTorch (version 1.7.1). Random data augmentation was performed, which included rotation, translation, zoom, and axis flipping. Our experiments used binary cross entropy with logits and mean squared error as loss function for the classification and regression networks, respectively, trained using the Adam optimizer [21]. The classification network was trained for up to 25 epochs, with a batch size of 4 and a learning rate decay every 10th epoch by a factor ⌊epoch/10⌋ × 10. The SUVR regression network was trained for up to 1000 epochs with a batch size of 32. Validation loss and accuracy was monitored to choose the best epoch for each fold. All computations were performed on an IBM POWER9 server with four NVIDIA TESLA V100 GPUs.

### Statistical analysis

Each scan was classified as amyloid positive or negative using a predefined threshold of 0.5 on the average predictions across the five amyloid status classification CNNs. Furthermore, binarized categories (positive/negative) based on a standard threshold on rSUVR were created. The threshold was determined by performing conventional receiver operating characteristic (ROC) analysis with expert classification as the binary variable and using only the training patients (*n* = 872). The threshold was used on both reference and predicted SUVR for the test patients to determine the SUVR-driven amyloid status accuracy. The predicted SUVR was determined by the median of the five network outputs. Measures of agreement (Cohen’s kappa), accuracy ((TP + TN)/(TP + TN + FP + FN)), sensitivity (TP/(TP + FN)), specificity (TN/(TN + FP)), and F1-score (2 * TP/(2 * TP + FP + FN)) were calculated for the validation and each of the hold-out test-sets independently, where TP = True Positive, TN = True Negative, FP = False Positive, and FN = False Negative. The ability to predict the SUVR value were compared against the reference by computing the coefficient of determination (*R*^2^).

### Benchmarking with public data

To provide a benchmark for our method, [^11^C]PiB PET/CT data (*n* = 224) were obtained from the ADNI database. The ADNI was launched in 2003 as a public–private partnership, led by Principal Investigator Michael W. Weiner, MD. The primary goal of ADNI has been to test whether serial magnetic resonance imaging, positron emission tomography, other biological markers, and clinical and neuropsychological assessment can be combined to measure the progression of mild cognitive impairment (MCI) and early Alzheimer’s disease. For up-to-date information, see http://www.adni-info.org. The PET images from the ADNI database originated from different scanners, and were obtained using the co-registered, averaged, and standardized image and voxel size PET pre-processing applied. We computed rSUVR using the same method as described in “Visual interpretation of amyloid PET and SUVR calculation” Section and evaluated the performance of our method using the same metrics as introduced in “Statistical analysis” Section. We did not perform a visual reading of the ADNI data, and thus, the reference amyloid status is purely based on rSUVR.

## Results

### Expert reading and reference SUVR

**Fig. 3:**
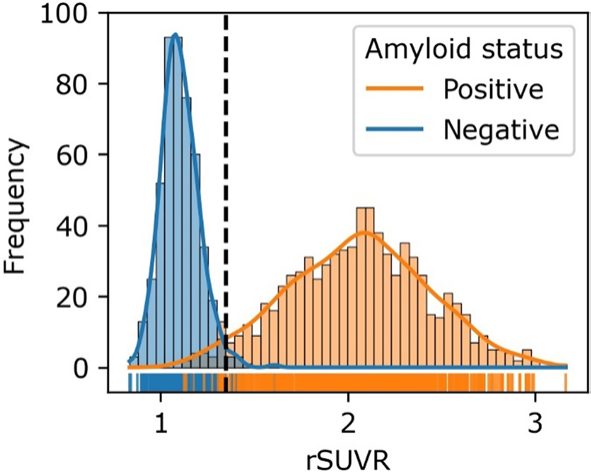
Distribution of rSUVR values for the entire cohort (*n* = 1309). The SUVR threshold (1.35) for binary classification into amyloid positive/negative read status is illustrated with a vertical dashed line. The threshold was determined using only the 872 training/validation subjects

Expert reading resulted in 61% of scans to have increased amyloid deposition; 62% prevalence in train/validation group, 62% in hold-out PET/CT group, and 46% in hold-out PET/MRI group. Figure 3 illustrates the distribution of rSUVR across all patients. The ROC analysis determined an optimal cut-off of 1.35 to categorize rSUVR into amyloid positive/negative read status based on the training/validation cohort. The agreement of rSUVR with the expert reader is shown for the two hold-out test cohorts in Table 2.

**Table 2:** Performance of an optimal semi-quantitative rSUVR threshold of 1.35 for categorization into amyloid positive/negative visual read status

	PET/CT	PET/MRI
	Hold-out test *n* = 300	Hold-out test *n* = 100
Cohen’s kappa	0.94	0.96
Accuracy	97%	98%
Sensitivity	97%	98%
Specificity	98%	98%
F1-score	98%	98%

### Classification model

**Fig. 4:**
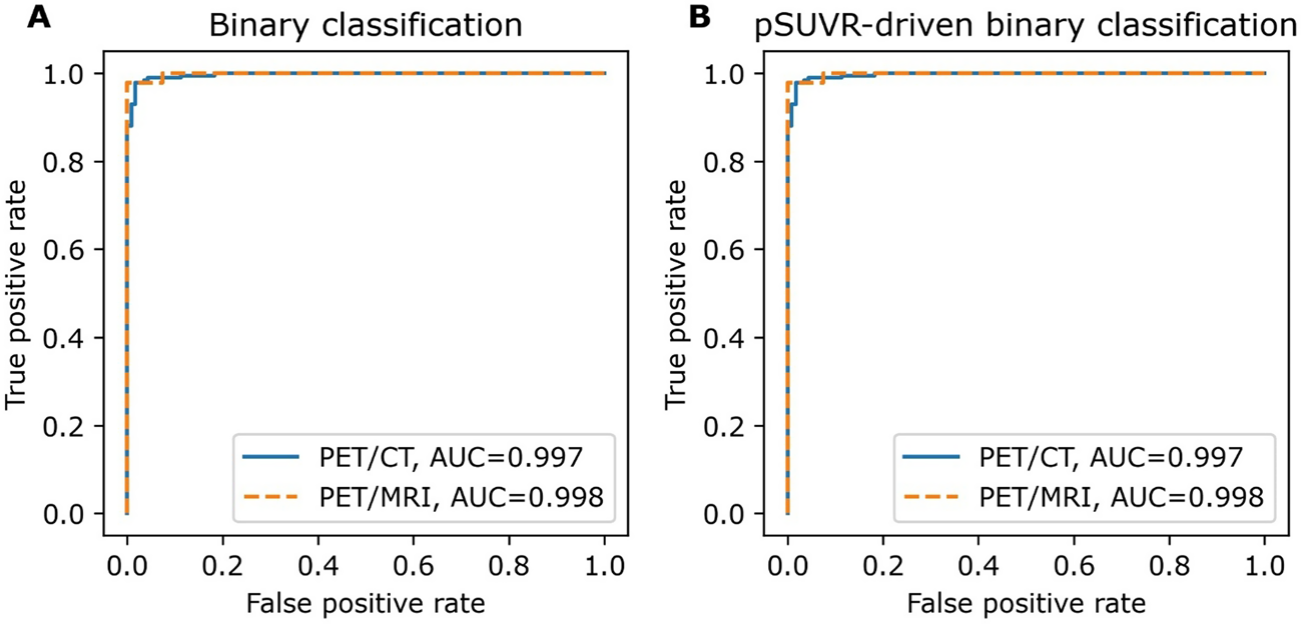
ROC curves for the hold-out test sets (*n* = 400). **A** show the result for the classification network against the expert reading. **B** show the result for the deep learning derived (regression network) pSUVR-driven amyloid status against the expert reading

The amyloid status classification model was trained using five-fold cross-validation, which each took up to 8 h to converge. The time to predict the diagnosis for a new patient was < 1 s. The proposed ensemble deep learning model correctly classified amyloid status in 296/300 (99%) of the hold-out PET/CT subjects, 4 were misclassified (3 false positive). The hold-out PET/MRI subjects were correctly classified in 98/100 (98%) of the cases, 2 was misclassified (1 false positive). Four of these misclassified subjects were borderline with rSUVR close to the cut-off of 1.35, one was affected by motion, and one had abnormally low activity in the image. The agreement with the expert reader classification is shown for the validation cohort and the two hold-out test cohorts in Table 3 and ROC curves are plotted for the hold-out test sets in Fig. 4A. Ensemble classification accuracy (99%, hold-out PET/CT) was an improvement over any of the individual predictions for each *k*-fold model (94–97%).

**Table 3:** Performance of the amyloid status classification deep learning model

	PET/CT		PET/MRI
	Validation *n* = 872*	Hold-out test *n* = 300	Hold-out test *n* = 100
Cohen’s kappa	0.96	0.97	0.96
Accuracy	98%	99%	98%
Sensitivity	98%	99%	98%
Specificity	97%	97%	98%
F1-score	98%	99%	98%

*The validation subjects represent the sum of all five validation folds (*n* = 872)

### Predicted SUVR

**Fig. 5:**
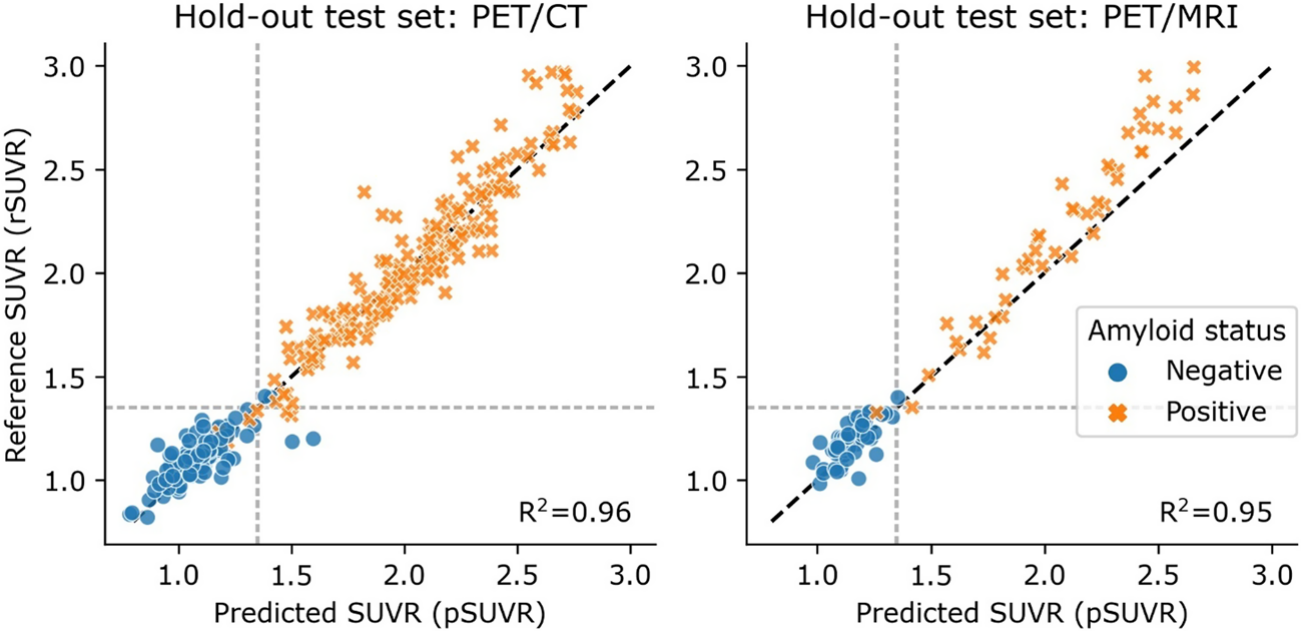
Correlation between reference and predicted SUVR for the PET/CT (left, *n* = 300) and PET/MRI (right, *n* = 100) hold-out test sets. The dotted gray lines illustrate the 1.35 threshold. The dashed black line is the identity line

The training time for the five regression networks was 14 h each, and again took less than 1 s to predict pSUVR for a new patient. The median of the five pSUVRs are shown against rSUVR in Fig. 5. The model successfully predicted values comparable to the rSUVR, as demonstrated by high coefficient of determination scores of 0.96 and 0.95 for the PET/CT and PET/MRI hold-out test sets, respectively. When applying the determined SUVR threshold of 1.35 for binary classification, amyloid status corresponded with the expert classification in all but 10 cases across both hold-out test cohorts for both the reference and predicted SUVR (Table 4). The ROC-curves are shown for the hold-out test sets in Fig. 4B. Strictly comparing SUVR-driven amyloid status of the predicted and reference SUVR, we found congruent classification in all but four cases in the hold-out PET/CT test set. The expert classification agreed with the rSUVR-driven status in two cases and with the pSUVR-driven status in the other two equivocal cases.

**Table 4:** Performance of the deep learning derived pSUVR-values to determine amyloid read status using a SUVR threshold of 1.35

	PET/CT	PET/MRI
	Hold-out test *n* = 300	Hold-out test *n* = 100
Cohen’s kappa	0.94	0.96
Accuracy	97%	98%
Sensitivity	98%	98%
Specificity	97%	98%
F1-score	98%	98%

### Competing pathology

The classification networks were able to correctly classify all but two subjects from the cohort of 37 patients with competing pathology and PET images characterized by a combination of anatomical distortions with borderline or atypical amyloid distribution (Additional file 1: Fig. S1). The SUVR-driven amyloid status agreed with the expert reader in all but 2 subjects (one different than above) using pSUVR and all but 3 subjects using the rSUVR.

### Benchmarking with public data

The classification networks agreed with the SUVR-driven amyloid status in all but 5 subjects (accuracy 98%, sensitivity 97%, specificity 100%) for the ADNI data. The regression networks were able to predict SUVR values with good correlation to the reference (Additional file 1: Fig. S2), which agreed with the reference SUVR-driven status in all but 8 subjects (accuracy 97%, sensitivity 99%, specificity 91%).

## Discussion

Development of disease modifying therapies directed toward the removal of amyloid deposits in AD may require fast and accurate classification methods in patient selection. Quantification of amyloid deposition is challenged by multiple processing steps and require structural MRI image segmentation to achieve highest accuracy [2, 3]. We developed end-to-end deep learning models that were able to classify amyloid status and semi-quantitative SUVR from only an amyloid PET image, pre-processed using an MRI or CT. It should be stressed that the predicted output was clinical reading, and not clinical outcome on follow-up evaluation or pathology. Our model reproduced the diagnosis made by an expert reader in two large independent test cohorts with high accuracy (99% accuracy compared to 97% when using standard SUVR method), and, thus, has the potential to support or replace visual reading in the future.

In general terms [^11^C]PiB amyloid PET imaging is very effective in separating patients into read positive and negative [22, 23], illustrated by our finding of 97% accuracy based on simple SUVR thresholding of 1.35. The positive read patients below this threshold (*n* = 7) were dominated by patients with intense uptake in just one region, which may be difficult to identify when several regions are weighted together as for SUVR, where the active region may simply not be measured or have an insignificant volume. Our model was able to further improve the already high accuracy to 99%, which could be due to application of a global evaluation that is not limited to a set of predefined regions where amyloid uptake typically is most pronounced. The global evaluation might further make the method less susceptible to changes in smaller regions caused by structural abnormalities, misregistration, motion, or atypical amyloid uptake. During quality assessment in routine clinical use, we have found the model useful to identify borderline cases that require more detailed reading based on divergent classifications on measured SUVR and AI.

Simply classifying amyloid status without providing measures quantifying the degree of pathology, challenges the usability of such a method in clinical routine. Thus, providing an estimate of the amyloid burden alongside the classification results in a higher confidence in the reached diagnosis [24]. The predicted or measured SUVR could also serve as a metric to prioritize resources for a more detailed reading of the patient. In practice there were no read negative patients above rSUVR of 1.61 or read positive patients below rSUVR 1.12, essentially defining an approximate SUVR range for borderline cases encompassing 23% of patients that require closer scrutiny by the reader.

The main strength of our model is that it was trained with PET data from multiple scanners, injected doses, real-life imaging guideline violations, reconstruction parameters, and resulting image noise characteristics and resolutions, which resulted in the model being highly generalizable. This was demonstrated by the PET/MRI hold-out test set where the model achieved 98% accuracy despite not having seen any PET/MRI data during training. Using patients from the ADNI database the accuracy was retained. These results, thus, suggest that our model is probably directly applicable across sites. The model furthermore appeared robust toward delayed acquisition, head motion, and pathology that affected the structural integrity of the brain, such as infarcts, hemorrhage, traumatic lesions, NPH and meningioma, and was trained and validated on a population, that reflect clinical patient throughput more accurately than the ADNI cohort. The model was also able to correctly classify the six patients in the test set that had received a dose less than 100 MBq. This indicates that the model has potential for ultra-low activity injection or fast acquisition imaging directly or after transfer learning. This needs to be confirmed in a separate study.

The largest deep learning amyloid PET study based on the ADNI database was proposed by Reith et al. [10] who utilized 2066 patients examined with [^18^F]Florbetapir. The model correlated well with MRI-derived SUVR but less with visual inspection by four expert readers. Compared to the model by Reith et al. that accepts three slices as input, our model accepts the full 3D volume, and is therefore not blinded to any parts of the brain. Kim et al. [14] further demonstrated that a model trained on [^18^F]Florbetapir PET data was transferable without loss of accuracy to an independent test cohort from the ADNI database imaged with [^18^F]Florbetaben without any retraining. Nai et al. [25] trained a model using [^11^C]PiB data partly from the ADNI database, and achieved a maximum accuracy of 95%, and de Vries et al. [11] similarly obtained an accuracy of 95% when using *n* = 22 [^18^F]Florbetapir patients from the ADNI database as test cohort. Only few studies exist based on local data. An example is Kang et al. [26] who obtained 92% accuracy with a slice-based classification model after applying gray matter masking in a small cohort of 176 patients. Validation on local data is important since models trained on public databases might not be transferable to local routine data, as outlined above.

Our study had several limitations. While using only a minimum of pre-processing, our method does rely on skull stripping and spatial normalization by affine registration. However, we found that local inaccuracies in either step did not appear to change the precision of the model. When developing the method, we investigated the impact of neglecting the spatial normalization, and found that this resulted in inferior performance (results not shown). Another limitation is the use of data solely from scanners by a single vendor and a single site. Despite appearing robust toward scanner type, it remains to be shown whether the model generalizes across vendors, radiotracers, and sites.

## Conclusion

We have trained a deep learning network capable of predicting amyloid status, agreeing with expert diagnosis in 99% of a mixed memory patient cohort in a large local dataset, while also predicting amyloid burden represented by the subjective semi-quantitative metric SUVR. This method has the potential to replace or simplify the existing clinical routine, can act as a tool for patient triaging, and can facilitate fast and accurate classification well-suited for a high throughput pipeline for patient selection for disease modifying therapy.

## Supplementary Information

**Additional file 1**: **Table S1**. PET acquisition and reconstruction information on included patients. **Table S2**. Hyper parameters for amyloid status classification network. The column “Options” indicates the range of values that was tested during hyper parameter search. N/A in the column indicates that the variable was fixed. **Table S3**. Hyper parameters for quantitative SUVR regression network. The column “Options” indicates the range of values that was tested during hyper parameter search. N/A in the column indicates that the variable was fixed. **Fig. S1**. Patients with co-morbidity and [11C]PiB PET amyloid read positive status misclassified with the deep learning image-based network as amyloid read negative. (A) Head CT and [11C]PiB PET (top row) from 69-year-old man with resection of right frontal meningioma performed 47 years previously leading to a large resection cavity, right hemisphere subdural hygroma, and hydrocephalus (red arrows). The resultant anatomical distortions made global SUVR calculations in standard regions inaccurate (rSUVR= 1.25), but the visual interpretation was amyloid read positive because of uptake in striatum, loss of gray/white matter contrast in left temporal lobe and mesial parietal lobes (green arrows). Statistic surface projections show distribution of lesions and cortical areas with uptake. (B) T2 weighted MRI FLAIR sequence and [11C]PiB PET (bottom row) from 69-year-old man with childhood head trauma after accident with resulting lesion and gliosis in frontotemporal area (red arrow). Atypical [11C]PiB PET distribution with amyloid read positive borderline uptake in left hemisphere predominantly in frontal lobe and striatum, and to a lesser degree in temporal lobe (green arrows). rSUVR = 1.59. **Fig. S2**. Correlation between reference and predicted SUVR for the ADNI hold-out test set. The dotted gray lines illustrate the 1.35 threshold. The dashed black line is the identity line.

## Data Availability

The datasets generated and/or analyzed during the current study are not publicly available due to it containing patient identifiable information. Requests to access these datasets should be directed at: ian.law@regionh.dk.

## Abbreviations

Aβ: Amyloid-β
AD: Alzheimer’s disease
ADNI: Alzheimer’s disease neuroimaging initiative
CNN: Convolutional neural network
FDA: Food and drug administration
MCI: Mild cognitive impairment
p.i.: Post-injection
PET: Positron emission tomography
PiB: Pittsburg-compound-B
pSUVR: Predicted standardized uptake value ratio
ROC: Receiver operating characteristic
rSUVR: Reference standardized uptake value ratio
SCD: Subjective cognitive decline
SUVR: Standardized uptake value ratio
T1w: T1-weighted

## Footnotes

1 https://clinicaltrials.gov/ct2/show/NCT00106899.
