# Supplementary material for: Estimation of brain amyloid accumulation using deep learning in clinical [11C]PiB PET imaging
Source: EJNMMI Phys. 2023 Jul 14;10:44. doi: 10.1186/s40658-023-00562-7 (PMC10348957; doi:10.1186/s40658-023-00562-7)
Supplement: Supplementary file 1 — Additional file 1: Table S1. PET acquisition and reconstruction information on included patients. Table S2. Hyper parameters for amyloid status classification network. The column “Options” indicates the range of values that was tested during hyper parameter search. N/A in the column indicates that the variable was fixed. Table S3. Hyper parameters for quantitative SUVR regression network. The column “Options” indicates the range of values that was tested during hyper parameter search. N/A in the column indicates that the variable was fixed. Fig. S1. Patients with co-morbidity and [11C]PiB PET amyloid read positive status misclassified with the deep learning image-based network as amyloid read negative. (A) Head CT and [11C]PiB PET (top row) from 69-year-old man with resection of right frontal meningioma performed 47 years previously leading to a large resection cavity, right hemisphere subdural hygroma, and hydrocephalus (red arrows). The resultant anatomical distortions made global SUVR calculations in standard regions inaccurate (rSUVR= 1.25), but the visual interpretation was amyloid read positive because of uptake in striatum, loss of gray/white matter contrast in left temporal lobe and mesial parietal lobes (green arrows). Statistic surface projections show distribution of lesions and cortical areas with uptake. (B) T2 weighted MRI FLAIR sequence and [11C]PiB PET (bottom row) from 69-year-old man with childhood head trauma after accident with resulting lesion and gliosis in frontotemporal area (red arrow). Atypical [11C]PiB PET distribution with amyloid read positive borderline uptake in left hemisphere predominantly in frontal lobe and striatum, and to a lesser degree in temporal lobe (green arrows). rSUVR = 1.59. Fig. S2. Correlation between reference and predicted SUVR for the ADNI hold-out test set. The dotted gray lines illustrate the 1.35 threshold. The dashed black line is the identity line. [file 40658_2023_562_MOESM1_ESM.docx]

**Supplementary Table 1:** PET acquisition and reconstruction information on included patients.

| **Scanner model** | **Scan duration** | **Matrix** | **Spacing** | **Filtering** | **Occurrences** | **Train** | **Test** |
| --- | --- | --- | --- | --- | --- | --- | --- |
| Biograph TruePoint | 20 min | 336 | 1.02x1.02x2.03 mm^3^ | 5 mm | 22 | 15 | 7 |
|  | 20 min | 336 | 1.02x1.02x3 mm^3^ | 5 mm | 883 | 631 | 252 |
|  | 30 min | 168 | 2.04x2.04x3 mm^3^ | 3 mm | 1 | 1 | 0 |
|  | 30 min | 166 | 2.04x2.04x3 mm^3^ | 4 mm | 1 | 1 | 0 |
|  | 30 min | 336 | 1.02x1.02x3 mm^3^ | 3 mm | 43 | 34 | 9 |
|  | 30 min | 336 | 1.02x1.02x3 mm^3^ | 4 mm | 9 | 8 | 1 |
|  | 30 min | 336 | 1.02x1.02x3 mm^3^ | 5 mm | 117 | 94 | 23 |
| Biograph mCT | 20 min | 400 | 1.02x1.02x2.03 mm^3^ | 5 mm | 4 | 2 | 2 |
|  | 20 min | 400 | 1.02x1.02x3 mm^3^ | 5 mm | 14 | 12 | 2 |
|  | 30 min | 400 | 1.02x1.02x3 mm^3^ | 4 mm | 3 | 3 | 0 |
| Biograph Vision | 20 min | 440 | 0.82x0.82x1.65 mm^3^ | 5 mm | 26 | 16 | 10 |
|  | 20 min | 440 | 0.55x0.55x3 mm^3^ | 5 mm | 62 | 43 | 19 |
|  | 20 min | 440 | 0.82x0.82x3 mm^3^ | 5 mm | 16 | 11 | 5 |
|  | 30 min | 440 | 0.55x0.55x3 mm^3^ | 5 mm | 1 | 1 | 0 |
| Biograph mMR | 20 min | 256 | 1.12x1.12x2.03 mm^3^ | 5 mm | 29 | 0 | 29 |
|  | 20 min | 344 | 0.83x0.83x2.03 mm^3^ | 5 mm | 24 | 0 | 24 |
|  | 30 min | 256 | 1.12x1.12x2.03 mm^3^ | 5 mm | 25 | 0 | 25 |
|  | 30 min | 344 | 0.83x0.83x2.03 mm^3^ | 5 mm | 29 | 0 | 29 |

**Supplementary Table 2:** Hyper parameters for amyloid status classification network. The column “Options” indicates the range of values that was tested during hyper parameter search. N/A in the column indicates that the variable was fixed.

| **Area** | **Hyper parameter** | **Options** | **Chosen** |
| --- | --- | --- | --- |
| Input | N/A | N/A | 256x256x256x1 |
| Block 1 | Convolution kernels | Discrete([3,5]) | 5 |
|  | Number of filters (C_1_) | Discrete([16,32]) | 32 |
|  | Dropout | RealInterval(0.1,0.5) | 0.13 |
| Block 2 | Convolution kernels | Discrete([3,5]) | 5 |
|  | Number of filters (C_2_) | Discrete([16,32]) | 32 |
|  | Dropout | RealInterval(0.1,0.5) | 0.14 |
| Block3 | Convolution kernels | Discrete([3,5]) | 3 |
|  | Number of filters (C_3_) | Discrete([16,32]) | 16 |
|  | Dropout | RealInterval(0.1,0.5) | 0.11 |
| Block 4 | Convolution kernels | Discrete([3,5,7]) | 3 |
|  | Number of filters (C_4_) | Discrete([16,32,64,128]) | 128 |
|  | Dropout | RealInterval(0.1,0.5) | 0.11 |
| Dense | # Features | N/A | 2,519,424 |
| N/A | Learning rate | RealInterval (1x10^-6^, 1x10^-3^) | 4x10^-4^ |

**Supplementary Table 3:** Hyper parameters for quantitative SUVR regression network. The column “Options” indicates the range of values that was tested during hyper parameter search. N/A in the column indicates that the variable was fixed.

| **Area** | **Hyper parameter** | **Options** | **Chosen** |
| --- | --- | --- | --- |
| Input | N/A | N/A | 128x128x128x1 |
| Block 1 | Convolution kernels | Discrete([3,5,7]) | 7 |
|  | Number of filters (C_1_) | Discrete([16,32,64,128]) | 32 |
|  | Dropout | N/A | 0.13 |
| Block 2 | Convolution kernels | Discrete([3,5,7]) | 7 |
|  | Number of filters (C_2_) | Discrete([16,32,64,128]) | 64 |
|  | Dropout | N/A |  |
| Block3 | Convolution kernels | Discrete([3,5,7]) | 5 |
|  | Number of filters (C_3_) | Discrete([16,32,64,128]) | 128 |
|  | Dropout | N/A | 0.11 |
| Block 4 | Convolution kernels | Discrete([3,5,7]) | 3 |
|  | Number of filters (C_4_) | Discrete([16,32,64,128]) | 256 |
|  | Dropout | N/A | 0.11 |
| Dense | # Features | N/A | 186,624 |
| N/A | Learning rate | N/A | 1x10^-4^ |

**
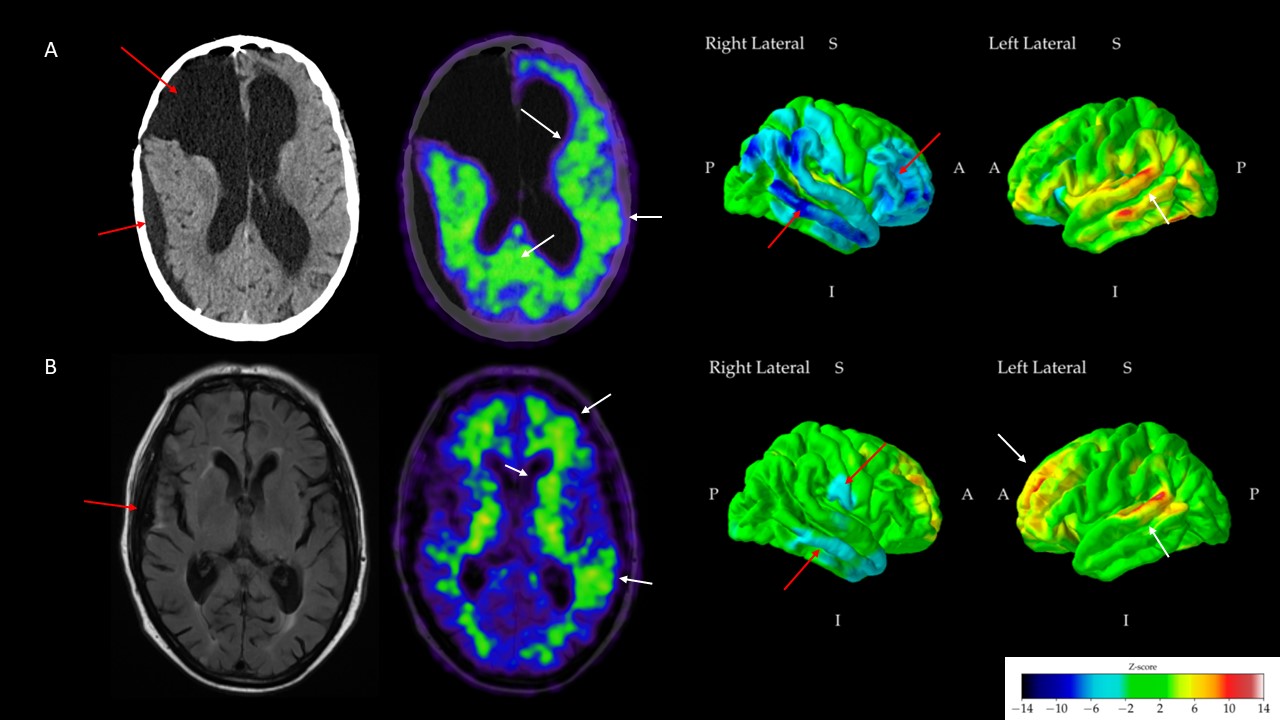
**

**Supplementary Figure 1:** Patients with co-morbidity and [^11^C]PiB PET amyloid read positive status misclassified with the deep learning image-based network as amyloid read negative. (A) Head CT and [^11^C]PiB PET (top row) from 69-year-old man with resection of right frontal meningioma performed 47 years previously leading to a large resection cavity, right hemisphere subdural hygroma, and hydrocephalus (red arrows). The resultant anatomical distortions made global SUVR calculations in standard regions inaccurate (rSUVR= 1.25), but the visual interpretation was amyloid read positive because of uptake in striatum, loss of grey/white matter contrast in left temporal lobe and mesial parietal lobes (green arrows). Statistic surface projections show distribution of lesions and cortical areas with uptake. (B) T2 weighted MRI FLAIR sequence and [^11^C]PiB PET (bottom row) from 69-year-old man with childhood head trauma after accident with resulting lesion and gliosis in frontotemporal area (red arrow). Atypical [^11^C]PiB PET distribution with amyloid read positive borderline uptake in left hemisphere predominantly in frontal lobe and striatum, and to a lesser degree in temporal lobe (green arrows). rSUVR = 1.59.

**
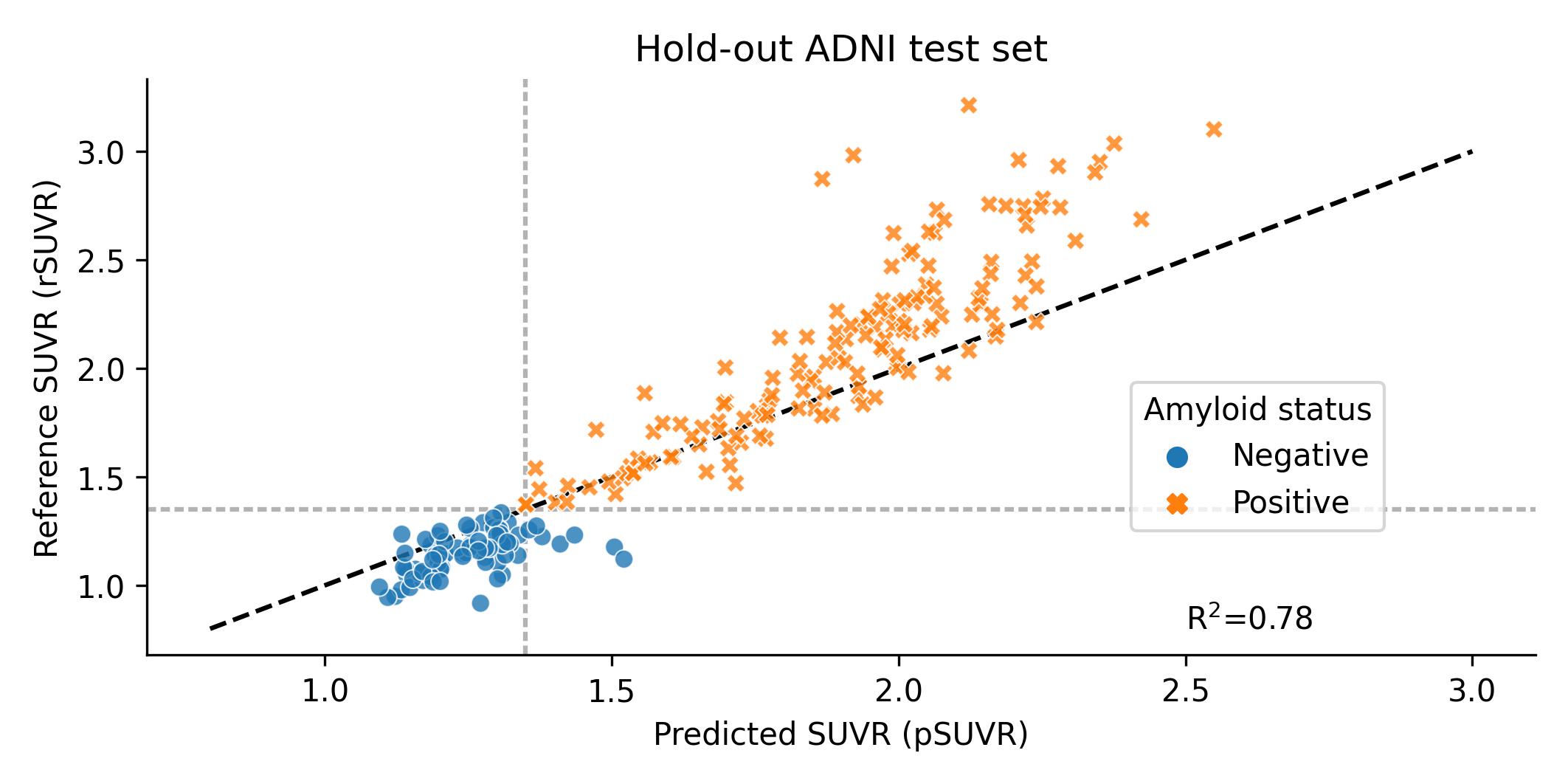
**

**Supplementary Figure 2:** Correlation between reference and predicted SUVR for the ADNI hold-out test set. The dotted gray lines illustrate the 1.35 threshold. The dashed black line is the identity line.
